# The value of contrast‐enhanced ultrasound in determining the location of sentinel lymph nodes in breast cancer

**DOI:** 10.1186/s40644-021-00397-4

**Published:** 2021-03-12

**Authors:** Jun Luo, Liting Feng, Qing Zhou, Qin Chen, Jinping Liu, Chihua Wu, Jing Luo, Jie Chen, Hao Wu, Wanyue Deng

**Affiliations:** 1 Department of Ultrasound, Sichuan Provincial People’s Hospital, University of Electronic Science and Technology of China, 32# W.Sec 2,1st Ring Rd, 610072 Chengdu, China; 2 Department of Breast Surgery, Sichuan Provincial People’s Hospital, University of Electronic Science and Technology of China, 610072 Chengdu, China

**Keywords:** Contrast‐enhanced ultrasound (CEUS), Breast cancer, Sentinel lymph node (SLN)

## Abstract

**Background:**

This study aimed to explore the sentinel lymph node (SLN) identification rate in breast cancer by subcutaneous and intradermal injection of ultrasound contrast agent in the mammary areola region, compared to the results achieved with methylene blue (MB).

**Methods:**

A total of 390 breast cancer patients with planned sentinel lymph node biopsy from our breast surgery department from July 2017 to February 2019 were enrolled. All patients were subjected to preoperative contrast-enhanced ultrasound (CEUS), that involved an intracutaneous injection of 1 mL ultrasonic contrast agent (UCA) at 3 and 6 o ‘clock, as well as a subcutaneous injection of 1 mL UCA at 9 and 12 o’clock. The enhanced lymph nodes along the enhanced lymphatic vessels from the mammary areola were traced. The number of enhanced lymph nodes were recorded, and an ultrasound-guided injection of 1:10 diluted carbon nanoparticles were used to mark all first site enhanced lymph nodes (i.e., SLNs). An intraoperative dye method (MB) was used to track the SLNs and the results were compared with the CEUS findings.

**Results:**

Among the 390 cases of breast cancer, enhanced SLNs were observed in 373 patients after an injection of UCA with an identification rate of 95.64 % (373/390), compared to the identification rate of 92.05 % (359/390) using the intraoperative MB. The difference between the two methods was statistically significant (*P* = 0.016). And among the 390 patients, a total of 808 enhanced lymph nodes were traced by preoperative CEUS, with a median of 2 (1,3). A total of 971 blue-stained lymph nodes were traced using the intraoperative MB, with a median of 2 (2,3), indicating a statistically significant difference (*p* < 0.001).

**Conclusions:**

Intradermal and subcutaneous injections of UCA in the mammary areola region may have clinical application value for the identification and localization of SLNs in breast cancer patients. The identification rate is higher than that of blue dye method, which can be used as a new tracer of sentinel lymph node biopsy and complement other staining methods to improve the success rate.

## Background

Breast cancer is the most common malignant tumor with the highest morbidity in women; however, breast cancer has a relatively good prognosis and prolonged survival time due to the development of recent treatment methods, such as radiotherapy, chemotherapy, endocrine therapy, and molecular targeted therapy [1]. Despite these methods, surgery remains the primary method of breast cancer treatment. Since the lymph nodes of the ipsilateral axilla drain the majority of the breast lymphatic drainage systems (> 75 %), the axillary lymph node (ALN) status is an important factor that affects the prognosis of breast cancer patients. It is known that malignant spread to ALN increases the 10-year recurrence rate from 20 %-30–70 % [2]. Therefore, the performance of an ipsilateral axillary lymph node dissection (ALND) during the traditional surgical procedures is necessary [3]. While a large number of studies have shown that patients with ALND have some sequelae, since ACOSOG Z0011 study less axillary surgery is being performed, and it is very important to identify the sentinel lymph node (SLN), so not to over treatment with the potential sequelae or undertreatment with potential recurrence [4, 5].

The SLN is the first site of mammary lymphatic drainage, and the risk of other lymph node metastasis is extremely low in the presence of SLNs without cancer metastasis. Moreover, patients without SLN metastasis can avoid ALND, which can improve their quality of life without having a significant impact on the long-term survival rate [6, 7]. Therefore, sentinel lymph node biopsy (SLNB) by “double dye”, especially blue-dye method combined with the isotopic tracer method,has become the standard surgical procedure for breast cancer patients with clinically node negative axilla [8]. However, the isotopic tracer method has not yet been applied to clinical work because of its radioactivity in China, so most hospitals adopt the single blue-dye method, which relies heavily on the experience of the surgeon.

Some studies [9, 10] have found that breast cancer patients have different lymphatic drainage patterns and number of SLNs, and contrast-enhanced ultrasound (CEUS) represents a feasible method of identifying the diversity of lymphatic drainage patterns and SLNs in breast cancer patients. This is because the UCA can freely enter the lymphatic capillaries across the lymphatic endothelial cell space and then drain to the lymph nodes. The SLN are the first enhanced lymph nodes that are traced along the enhanced lymphatic vessels. Previous studies searching for SLN via CEUS have failed to reach a consensus regarding the injection site and dose of UCA, and the SLN identification rate also remains highly variable. The UCA injection sites in previous studies include cubital vein, intra-tumor, peritumor, and areola injections. In addition, the injection depth includes intradermal, subcutaneous, and intra-gland injections, and the injection dose varies from 0.2 mL to 2.4 mL.

In this study, by referring to the previous literature on serching for SLN via CEUS, analyzing the influence of different injection sites and dose of UCA on the identification rate, a new injection method of UCA was developed to improve the identification rate of SLN by CEUS.

## Methods

### Patients

This study was approved by the ethics committee of Sichuan Academy of Medical Sciences & Sichuan Provincial People’s Hospital.

Patients diagnosed with malignant breast tumors by biopsy from the Department of Breast Surgery at Sichuan Provincial People’s Hospital were recruited to the present study. According to the SLNB indications mentioned in the guidelines and norms for the diagnosis and treatment of breast cancer by the Chinese anti-cancer association (2017) [8] and the appropriate application of CEUS, the specific inclusion criteria for this study were as follows: (1) patients with a malignant breast tumor; (2) negative for clinical axillary lymph nodes; (3) presence of single or multicentric lesions; (4) any age and sex; (5) plan to undergo a mastectomy and sentinel lymph node biopsy; (6) plan to undergo a breast conserving surgery and sentinel lymph node biopsy; (7) voluntarily accept the examination of CEUS-mediated tracking of SLNs and signing of informed consent. The specific exclusion criteria were: (1) inflammatory breast cancer; (2) severe lung function impairment; (3) allergy to the ultrasonic contrast agent; (4) a history of axillary surgery or radiotherapy; (5) distant metastasis of breast cancer.

### Methods

**Fig. 1:**
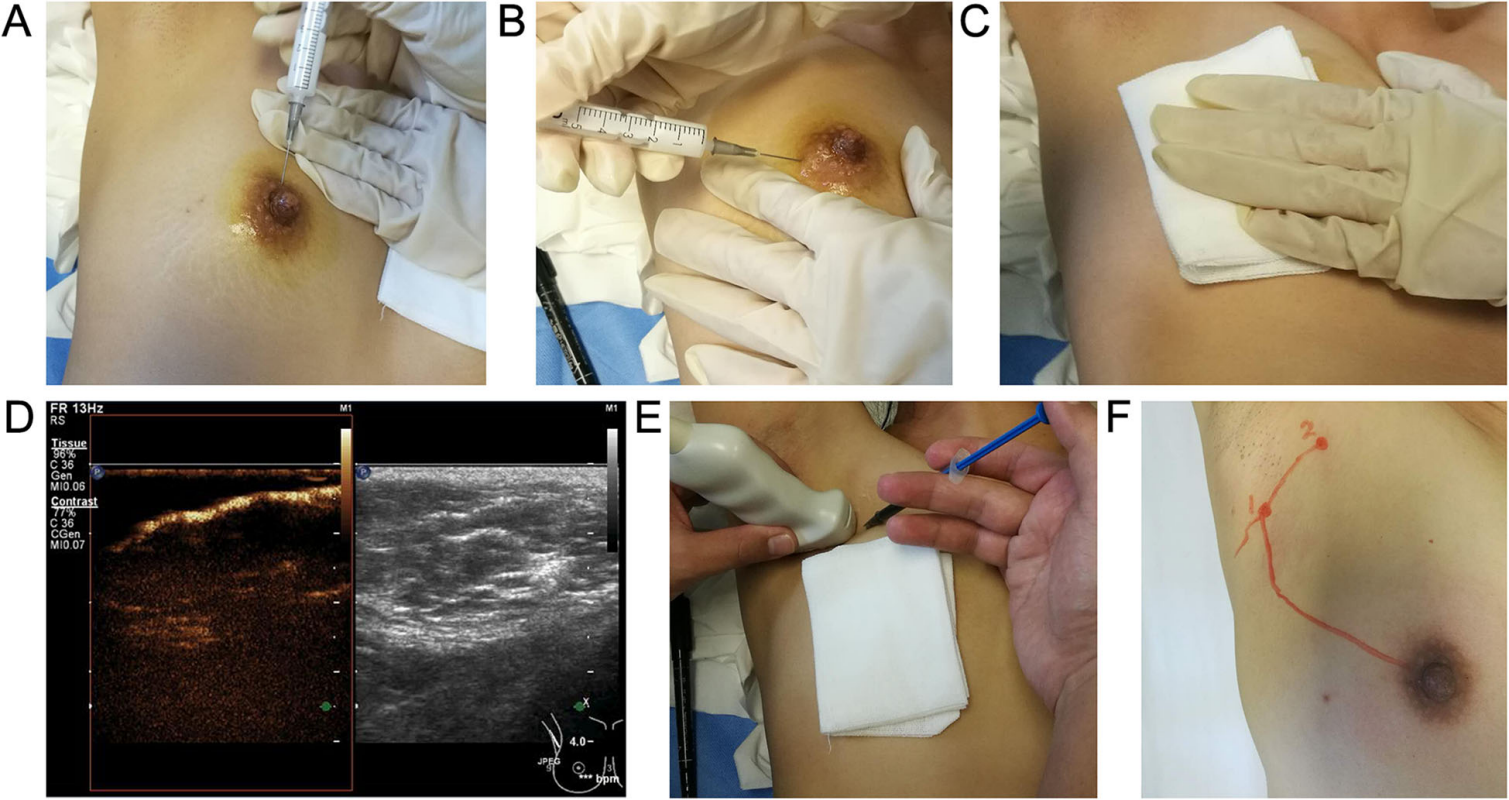
CEUS tracing the SLN. **a,b** An intradermal injection of 1 mL UCA at 3 and 6 o ‘clock and a subcutaneous injection of 1 mL UCA at 9 and 12 o ‘clock of the mammary areola region was performed following disinfection; **c** The injection site was then massaged gently to ensure adequate absorption of the UCA by the lymphatic vessels; **d **360° scanning of the areola area was performed to detect enhanced lymphatic vessels and trace the enhanced lymph nodes along the enhanced lymphatic vessels; **e** 0.1ml prepared 1:10 diluted carbon nanoparticles was injected into the first SLN site in CEUS; **f** The lymphatic vessel routes as well as the lymph node location were marked using a marking pen

All of the patients received CEUS the day before or the day of surgery using a Philips iU Elite. The assistant first extracted 5 mL of normal saline, which was injected into the UCA (59 mg SF6) and was fully mixed prior to the injection. Then, 0.9 mL normal saline and 0.1 mL carbon nanoparticles were extracted to prepare the dyes for future injection. Each patient was placed in the supine position, the upper limb of the affected side was lifted up into the same position as the intraoperative position, and the breast and axilla of the affected side was fully exposed. An intradermal injection of 1 mL UCA at 3 and 6 o ‘clock and a subcutaneous injection of 1 mL UCA at 9 and 12 o ‘clock of the mammary areola region was performed following disinfection. The injection site was then massaged gently until the lymphatic vessels began to enhanced to ensure adequate absorption of the UCA by the lymphatic vessels. A Philips iU Elite was set to contrast-mode, and 360° scanning of the areola area was performed to detect enhanced lymphatic vessels and trace the enhanced lymph nodes along the enhanced lymphatic vessels with a L9-3 high frequency linear array probe. 0.1ml prepared 1:10 diluted carbon nanoparticles was injected into the first SLN site in CEUS, and the number of lymph nodes traced by CEUS were recorded and the lymphatic vessel routes as well as the lymph node location were marked using a marking pen (Fig. 1). All CEUS examinations were performed by an experienced sonographer who had been engaged in breast ultrasound and intervention ultrasound for more than five years. All of the patients who underwent CEUS received a multi-point injection with MB subcutaneously and intradermally around the areola region 15 min before surgery. At surgery, after incising the skin, the dyed lymph nodes were traced along the dyed lymphatic vessels, and the results were compared with that of CEUS. The SLNs detected by CEUS and MB, as well as the enlarged lymph nodes in adjacent regions were all sent for pathological biopsy.

### Statistical analysis

SPSS17.0 was used for data processing. The measurement data were expressed as`x ± s if they were normally distributed, and the median (M) and upper and lower quartiles (P25, P75) were used if they were not normally distributed. The identification rate was defined by the proportion of patients with SLNs identified using either method. The comparison of the SLN identification rate between the two methods was subjected to a χ^2^ test. The level of significance was set at *P* < 0.05. If the number of lymph nodes detected by the two methods conformed to normal distribution, the paired sample T test was used for comparison; if not, the paired sample rank sum test was used. The level of significance was set at *P* < 0.05.

## Results

A total of 390 breast cancer patients from our breast surgery department from July 2017 to February 2019 were analyzed in the present study. Table 1 presents the histology results. All of the patients were female, aged from 22 to 85 years old, with a median age of 49(43,60). All the lesions were single, with a maximum diameter of 0.5 cm − 6.0 cm.

**Table 1:** The histology of 356 breast cancer patients

Histology	Number
Invasive breast cancer	275
Ductal carcinoma in situ	71
Intraductal papillary carcinoma	16
Mucinous carcinoma	10
Tubular carcinoma	2
Adenoid cystic carcinoma	1
Secretory carcinoma	1
Solid papillary carcinoma	4
Malignant phyllodes tumor	6
Borderline lobe tumor	4
Total	390

**Fig. 2:**
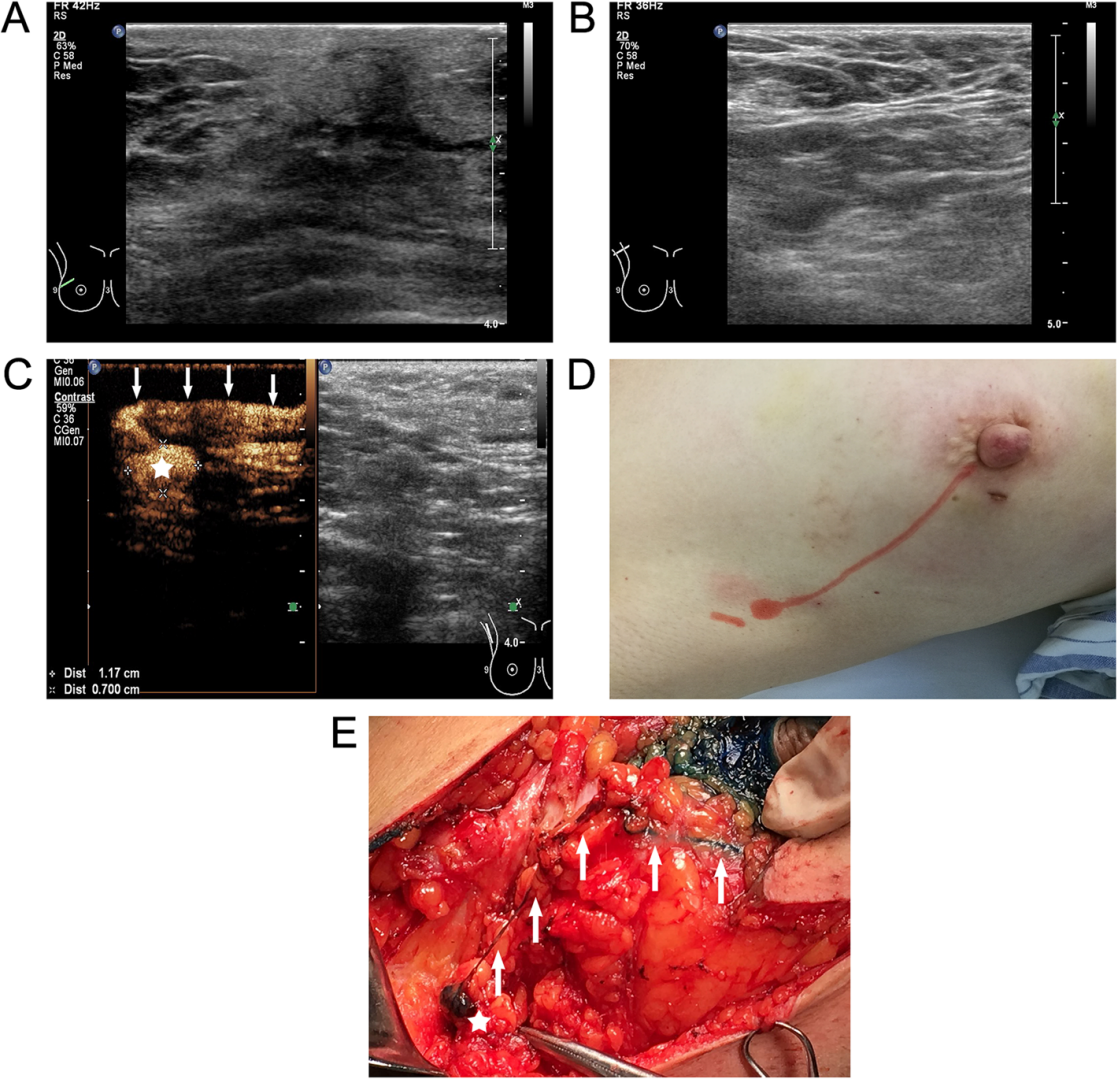
Case1, 54 year-old woman, Ductal carcinoma in situ. **a** B-Mode Ultrasonography of right external upper quadrant ductal carcinoma in situ; **b** B-Mode Ultrasonography of the right axillary; **c** traced the enhanced SLN (asterisk) along the enhanced lymphatic vessel (arrows) after injecting the UCA; **d**The body surface projection of enhanced lymphatic vessel and lymph node; **e**Traced the dyed SLN (asterisk) along the dyed lymphatic vessel (arrows) after injecting the MB before surgery, the lymphatic drainage pattern traced by CEUS was consistent with MB

**Fig. 3:**
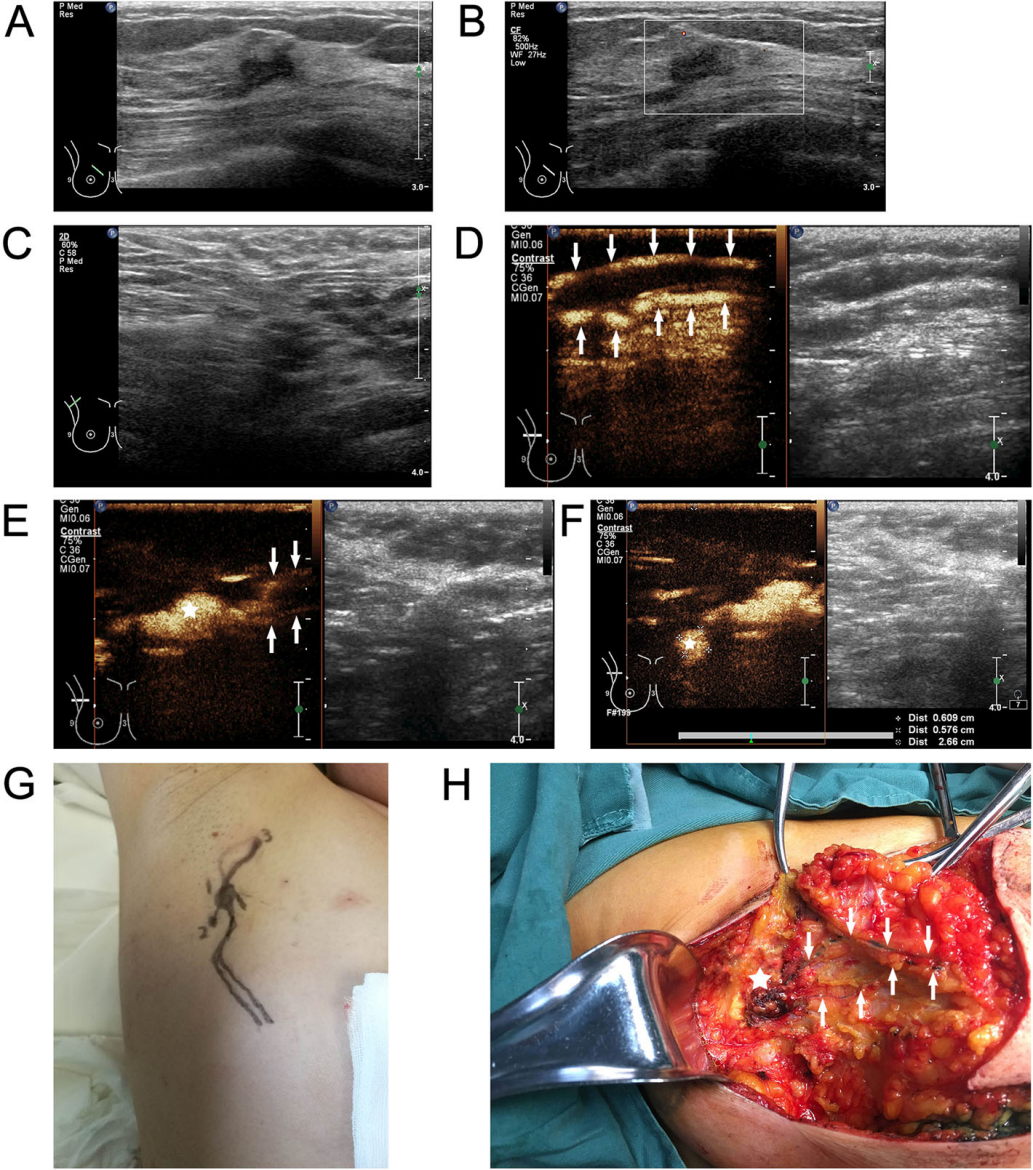
Case2, 52 year-old woman, Invasive ductal carcinoma.**a** B-Mode Ultrasonography of right inner upper quadrant invasive ductal carcinoma; **b** Color-Doppler Ultrasonography of right inner upper quadrant invasive ductal carcinoma; **c** B-Mode Ultrasonography of the right axillary; **d**Traced two enhanced lymphatic vessel (arrows) after injecting the UCA; **e** Both lymphatic vessel (arrows) drain to the same enhanced SLN (asterisk); **f**UCA can continue to drain to subsequent enhanced lymph node (asterisk); **g** The body surface projection of enhanced lymphatic vessel and lymph node; **h**Traced two dyed lymphatic vessel (arrows) drain to the same enhanced SLN (asterisk) after injecting the MB before surgery, the lymphatic drainage pattern traced by CEUS was consistent with MB

Among the 390 cases of breast cancer, enhanced SLNs were observed in 373 patients after the injection of UCA, with an identification rate of 95.64 % (373/390). And the identification rate of the intraoperative dye method was 92.05 % (359/390), The difference between the two methods was statistically significant (*P* = 0.016). The details of these identification are shown in Tables 2 and 3. And whether the SLNs had metastases are shown in Table 3. (The cases of CEUS vs. MB are shown in Figs. 2 and 3).

**Table 2:** The SLN identification rate by CEUS and MB

CEUS	MB		Total	
	+	-		
+	351	22	373	
-	8	9	17	
Total	359	31	390	^※^*P* = 0.016

^※^Compared with the identification rate of SLN traced by CEUS and MB, a χ^2^ test was used, and the difference was statistically significant (*P* = 0.016)

**Table 3:** The classification of SLNs according to CEUS and Methylene blue (MB)

Classification	Number of cases	Number of positive cases	Number of negative cases
^a^CEUS^+^/^b^MB^+^	351	61	290
CEUS^+^/^d^MB^−^	22	9	13
^c^CEUS^−^/MB^+^	8	0	8
CEUS^−^/MB^−^	9	8	1
Total	390	78	312

^a^ enhanced SLNs were observed by CEUS^b^ Blue-stained SLNs were observed by MB^c^ enhanced SLNs were not observed by CEUS^d^ Blue-stained SLNs were not observed by MB

A total of 808 enhanced lymph nodes were traced in CEUS, with a median of 2 (1,3). And 971 blue-stained lymph nodes were traced by intraoperative injection of MB, with a median of 2 (2,3), which could observe significantly more lymph nodes (*P* < 0.001). Please refer to Table 4 for the specific number of lymph nodes.

**Table 4:** The number of lymph nodes per patient traced by CEUS and MB

no. lymph nodes	The number and percentage of patients				
	CEUS	Percentage (%)	MB	Percentage (%)	
0	17	4.4	31	7.9	
1	120	30.8	26	6.7	
2	132	33.8	155	39.7	
3	71	18.2	106	27.2	
4	39	10.0	43	11.0	
5	11	2.8	29	7.4	
Total	390	100.0	390	100.0	^※^*p* < 0.001

^※^Compared with the number of lymph nodes traced by CEUS and MB, the data did not conform to a normal distribution, and the paired sample rank sum test was used. *P* < 0.001, the difference was statistically significant. MB could observe more lymph nodes

## Discussion

Grade 1 evidence of evidence-based medicine (EBM) shows that SLNB can safely and effectively replace ALND in patients without SLN metastasis, and the survival rate of SLNB was not worse than that of ALND after receiving standardized adjuvant therapy, dispite one or two SLNs metastasis [11, 12]. The key purpose of SLNB is to accurately identify and locate SLNs so as to reduce the potential false negative rate.

Some studies have found that CEUS has the potential to identify SLNs, While these studies have failed to reach a consensus regarding the injection site and dose of UCA. Some of the studies have shown that intra-tumor, peritumor, and areola injections have similar identification rate of SLNs [13, 14]. Some other studies and meta-analysis suggested that areola injection has a higher identification rate of SLNs [15, 16]. Moreover, an intradermal or subcutaneous injection of UCA is associated with a greater identification rate of SLNs compared to an injection into the mammary gland, which is due to the higher density of the superficial lymphatic network. By referring to the previous literature, in this study, an intradermal injection of 1 mL UCA at 3 and 6 o ‘clock, as well as a subcutaneous injection of 1 ml UCA at 9 and 12 o ‘clock into the mammary areola region was performed. This method was used to ensure that the lymphatic vessels under the areola region fully absorb the UCA and drain to the sentinel node area, the identification rate of the CEUS method was 95.64 % (373/390).

There were 9 out of 17 patients who did not have enhanced SLN with CEUS, in which interrupted lymphatic vessels and no dyeing of the lymph node was observed using the intraoperative dye method, 8 cases of the pathological results from ALND revealed ALN with cancer metastasis. It is possible that metastatic cancer cells may completely obstruct the lymphatic vessels, resulting in the inability of UCA and MB to drain to the lymph nodes [17]. The dyeing of lymph nodes using the intraoperative dye method and the pathological results were observed to be benign in the other 8 patients. Moreover, each of these patients had large tumors or hematoma following a puncture in the outer upper quadrant, which can compress the lymphatic vessels and affect the absorption and drainage of the UCA. This observation may be related to the different molecular weights of the tracers and physiological mechanisms of lymphatic absorption. It may also be that the lymphatic vessels only absorb a small amount of UCA, resulting in a weak lymphatic enhancement that is overlooked.

There were 351 cases out of the 373 patients in which enhanced SLNs observed by CEUS displayed dyeing of the lymph nodes using the intraoperative dye method. In another 22 cases, interrupted lymphatic vessels and no dyeing of the lymph node was observed with the intraoperative dye method, of which 9 cases displayed lymph node metastasis. Thus, CEUS can identify some SLNs that cannot be detected using the dye method. This is consistent with the findings of Xie et al., [18] in which patients who were not tracked for SLN staining with the intraoperative blue-dye method received CEUS examination before surgery. Thus, these patients received SLNB conditionally, avoiding the deficiency of the single dye method.

Regarding the number of observed lymph nodes, more lymph nodes were observed with MB than CEUS, which is consistent with the results of some previous studies [2, 18–21]. Since MB has a small molecular weight and lymphatic vessels have good absorbency of MB, when it drains to the SLN after injection, there is partial drainage to subsequent lymph nodes other than the SLNs, causing some of the subsequent lymph nodes to be stained blue during the process of intraoperative detection. However, the dye method requires that all stained lymph nodes to be sent for pathological biopsy, and there is currently no limitation on the number of lymph nodes, which may lead to the removal of too many non-sentinel lymph nodes and loss of SLNB significance. CEUS, on the other hand, is a real-time and dynamic imaging method. With the continuously tracking of enhanced lymph nodes along the enhanced lymphatic vessels immediately after the injection of UCA, some deep or unenhanced subsequent draining lymph nodes were not detected. In some cases, enhanced subsequent lymph node can be observed, but the first site of enhanced lymph nodes and the subsequent lymph nodes can be identified more readily by observing the time of UCA arrival and its connection to the lymphatic vessels.

Previous reports have shown that the identification rate of SLN by CEUS was 70 %~100 % [21]. The injection site and dose of UCA are inconsistent, and the SLN localization methods are diversified. Table 5 lists some of the literature reports with relatively high identification rate of SLN [18–20, 22–24]. Liu et al. [24] and Zhong et al. [19] had 9 and 8 cases, respectively, of presumed SLNs located by conventional ultrasound and interrupted enhanced lymphatic vessels, which may result in their higher identification rates than they actually were. Li et al. [23] observed that 85.4 % of lymphatic vessels began at upper outer quadrant, 14.6 % lymphatic vessels at other quadrants. Thus, the injection site of UCA should be in four quadrants of the areola to avoid missing enhanced SLNs. In our study, 8 patients failed to observe SLNs by CEUS, which had large tumors or hematoma following a puncture in the outer upper quadrant. And Xie et al. [18] chose the outer edge of the incision as the injection site instead if an incision was performed in upper outer quadrant previously. Multiple injection sites may improve SLN identification rate, but there is no consensus on the optimal injection dose. The body surface location alone was not accurate relatively due to the high activity of mammary gland, SLNs should be marked with more precise methods such as guidewire or staining.

**Table 5:** Characteristics of included studies

Author, year	Patients (n)	Injection site	Injection dose	Identification rate	Number of SLNs	Localization method
Liu,2019 [24]	75	Intradermally inject at 3,6,9,12 o’clock around the arela	0.5ml x 4	94.67 % (71/75)	116	Skin marked
Li,2019 [23]	453	Intradermally inject at 3,6,9,12 o’clock around the arela	0.6ml x 4	98.2 % (445/453)	765	Skin marked
Kenzo,2019 [22]	75	Intradermally and subdermally inject at 3,6,9,12 o’clock around the arela	0.25ml x 8	100 % (100/100)	92	Skin marked
Zhong,2018 [19]	126	Intradermally inject at outer upper quadrant near the areola	2ml	100 % (126/126)	164	FNA
Xie,2015 [18]	101	Intradermally inject at outer upper quadrant near the areola	1.5ml	97.03 % (98/101)	115	Guidewire
Zhao,2018 [20]	110	Intradermally inject at the periareolar area	0.4ml (One or two additional injections if failed)	96.4 % (106/110)	134	Skin marked

MB is prone to allergic reactions, local skin flap and adipose tissue necrosis in previous study [21], and one patient developed localized skin necrosis in our study. No patients had adverse reaction to CEUS in this study.In addition, CEUS can display lymphatic vessels and lymph nodes visually. The lymphatic route and SLNs position in the body surface is marked using a marker pen, which can help surgeons to more accurately select the surgical incision, identify lymphatic vessels and SLNs more easily. Moreover, 0.1ml prepared diluted carbon nanoparticles was injected into the SLNs in our study, to ensure that the black lymph nodes which the surgeon excise were the SLNs that were traced by CEUS.There are some limitations associated with this study. Due to the injection of UCA into the areola area, some patients with pain sensitivity experienced high levels of pain. Additionally, due to the control of domestic nuclides, the patients in this study could not track SLNs via nuclide tracing, and could only be compared using the dye method. Previous literature [18, 20] has shown that the identification rate of SLN can be improved by injecting the UCA again or injecting the UCA at the outer edge of the incision in the outer upper quadrant but we didn’t do that in our research, that’s something we can improve on.

## Conclusions

Intradermal and subcutaneous injections of UCA in the mammary areola region may have clinical application value for the identification and localization of SLNs in breast cancer patients. And it can identify some SLNs which cannot be identified using the dye method, which can be used as a new tracer of SLNB and complement other staining methods to improve the success rate.

## Data Availability

The datasets used and/or analyzed during the current study are available from the corresponding author on reasonable request.

## Abbreviations

ALN: Axillary lymph node
ALND: Axillary lymph node dissection
CEUS: Contrast-enhanced ultrasound
EBM: Evidence-based medicine
MB: Methylene blue
SLN: Sentinel lymph node
SLNB: Sentinel lymph node biopsy
UCA: Ultrasonic contrast agent
